# Conflict of Interests in the Scientific Production on Vitamin D and COVID-19: A Scoping Review

**DOI:** 10.3389/fpubh.2022.821740

**Published:** 2022-07-11

**Authors:** Carolina Saggioro Meissonier Passini, Maria Birman Cavalcanti, Simone Augusta Ribas, Camila Maranha Paes de Carvalho, Cláudia Bocca, Fernando Lamarca

**Affiliations:** ^1^School of Nutrition, Federal University of the State of Rio de Janeiro (UNIRIO), Rio de Janeiro, Brazil; ^2^Department of Public Health Nutrition, Federal University of the State of Rio de Janeiro (UNIRIO), Rio de Janeiro, Brazil; ^3^Graduate Program in Food and Nutrition Security (PPGSAN), Federal University of the State of Rio de Janeiro (UNIRIO), Rio de Janeiro, Brazil; ^4^Department of Social Nutrition, Fluminense Federal University (UFF), Rio de Janeiro, Brazil; ^5^Department of Applied Nutrition, Federal University of the State of Rio de Janeiro (UNIRIO), Rio de Janeiro, Brazil; ^6^Department of Applied Nutrition, Rio de Janeiro State University (UERJ), Rio de Janeiro, Brazil

**Keywords:** conflict of interest (COI), COVID-19, public health, SARS-CoV-2, vitamin D, scientific production, corporations, commercial determinants of health

## Abstract

The use of scientific evidence to support the process of formulating and implementing public policies might be biased by studies funded by the pharmaceutical and food industry, which more often than not meet corporate interests. This review aimed to analyze the occurrence of conflict of interest (COI) in academic production regarding vitamin D and COVID-19, considering the facility offered during the pandemic for academic publications of heterogeneous quality. A scoping review of observational studies published in Medline, Lilacs, and Google Scholar databases was carried out. The selected studies were published between December 2019 and August 2021, focused on the relationship between vitamin D and prevention or treatment of COVID-19 in non-institutionalized individuals, with no language restrictions. Twenty-nine studies met eligibility criteria. COI was disclosed in five papers and further identified by review authors in eight other papers, meaning COI was present in thirteen papers (44.8%). Studies were funded by companies in the diagnostics, pharmaceutical and food sectors. Conclusions favorable to vitamin D supplementation were more prevalent in papers where COI was identified (9/13, 69.2%) than among papers where COI was not found (4/16, 25.0%). Omissions of disclosure of COI, funding source, and sponsor functions were observed. The identification of possible corporate political activities in scientific papers about vitamin D published during the COVID-19 pandemic signals a need for greater transparency and guideline development on the prevention of COI in scientific production.

## Introduction

In the end of 2019, the SARS-CoV-2 virus emerged in China, causing first a local epidemic and soon spreading around the world, becoming one of the greatest challenges in public health of the XXI century (1). As scientific publications related to COVID-19 grew in volume over a short period of time, studies of heterogeneous quality were published (2), and gained prominence in academic platforms and mainstream media—often without taking into account their limitations or methodological weaknesses.

In this context, some studies in the field of nutrition and dietetics sought to relate the incidence or severity of COVID-19 to deficiency or supplementation of specific nutrients (3), including vitamin D (4). Public interest about the use of vitamins increased dramatically, as indicated by analyses of Google Trends data (5). Meanwhile, in Brazil, where vitamin D supplements are sold over the counter, the Federal Council of Pharmacy registered a two-fold increase in sales of cholecalciferol between April 2020 and April 2021 (6).

Conflict of Interest (COI) as a concept has been increasingly debated in health research, education and practice (7). COI are defined in medical research as circumstances that create a risk that professional judgments or actions regarding a primary interest (e.g., promoting and protecting the integrity of research) could be unduly influenced by a secondary interest (e.g., financial interest) (8, 9). Within food and nutrition, the debate around the participation and influence of the food and pharmaceutical industry in scientific production is also growing (10, 11). Companies often seek to defend their own interests by funding scientific studies that could benefit them and might influence the process of formulating and implementing public policies (12, 13). This is one of the strategies used by food industries presented in the seminal paper by Mialon et al. (12) that identified and defined so-called Corporate Political Activities (CPA).

Within the category of strategies linked to the manipulation of information, CPA include: shaping scientific agenda related to food and public health through investments in academic research funds; “cherry picking”, which consists in selecting only favorable findings; dissemination of unpublished research or research that had not been peer-reviewed; providing sponsored educational materials; supplying or influencing the dissemination of favorable research; valuing disagreement among experts and emphasizing doubt and uncertainty in science; among other actions (12). Evidence suggests that industry-sponsored researchers tend to publish research results and give out advice which are more favorable to their sponsors' products (14–17).

Given the influence of corporations on academic production and the opportunities for publishing less rigorous studies during the pandemic, the identification of COI in scientific findings seems more important than ever. A better understanding of COI in academic papers is relevant both to guide professional practice (2, 8) and to foment recognition and debate regarding this subject among researchers and authors of articles since problems with COI reporting in papers published in biomedical journals persist nowadays (18). It is unclear which CPA strategies can be identified in these heterogeneous studies, the level of transparency adopted by the authors regarding the relationship with vitamin D-related corporations, the COI and sources of funding disclosures, and the behavior of the results. For these reasons, the design of a scoping review was adopted to map the studies produced in this area during the COVID-19 pandemic, as well as to identify gaps in existing knowledge.

Thus, the aim of this review is to identify CPA present in observational studies that relate to vitamin D to COVID-19 infections. In order to do that, we seek to establish if COI related to pharmaceutical- and food- industries funding is associated with the recommendation of vitamin D supplementation. The present study does not intend to define whether vitamin D supplementation could bring potential benefits for the prevention or treatment of COVID-19.

## Materials and Methods

### Protocol

The methodological framework of this study was based on the recommendations for scoping reviews (19) and the findings were reported according to the Preferred Reporting Items for Systematic Reviews and Meta-Analysis extension for Scoping Reviews (PRISMA-ScR) recommendations (20).

The construction of the title, research question, and inclusion criteria was guided according to the JBI Manual for Evidence Synthesis (21) and based on the mnemonic Population/Participants, Concept, and Context (PCC) are described below.

Population/Participants: scientific production of vitamin D in non-hospitalized individuals.

Concept: Conflict of interests.

Context: prevention or treatment for COVID-19.

### Review Question

What CPA can be found in observational studies on vitamin D and COVID-19?

### Eligibility Criteria

This review included observational studies published between December 2019 and August 2021 that reported or evaluated the use of vitamin D as a strategy for prevention or treatment for SARS-CoV-2 infection among non-hospitalized individuals. This profile of individuals differs from those hospitalized, who may have other levels of impairment and different nutritional needs from the general population. There was no sex or age restriction. Also, there was no language restriction, however, descriptors were typed in in English and Spanish.

Review studies, letters, conference abstracts, opinion articles, books, case reports, clinical trials, *in vivo* (animal) and *in vitro* experimental studies were excluded, as well as observational studies that exclusively assessed hospitalized patients or not peer-reviewed. We chose to review observational studies because of their unique characteristics. Such designs, while not as methodologically robust as randomized clinical trials, are often employed to generate hypotheses about causality, which should be later tested with more rigorous research. Since observation studies raise less ethical issues and are generally less expensive and faster than intervention studies, they are abundant. It is also worth noting that they can go from conception to publication quickly, especially when employing secondary data analysis. Therefore, observational studies with and without COI were being published since the very beginning of the pandemic.

### Information Sources and Search Strategy

Searches were conducted on August 24, 2021 on three open access electronic databases: Medline, Latin American and Caribbean Health Sciences Literature (Lilacs), and Google Scholar (restricted to the first 200 references). In addition, manual searches were performed, using the reference list of articles found seeking other potentially eligible studies.

The search strategy was developed according to the criteria established by the Peer Review of Electronic Search Strategies (PRESS checklist) (22). An external researcher, specialist in systematic review in the area of food and nutrition, evaluated and contributed to its adequacy. The search strategy was adapted to each platform used from this one: [(“vitamin D” OR “vitamin d2” OR “vitamin d3” OR “cholecalciferol” OR “ergocalciferol” OR “calcitriol” OR “25-hydroxy-cholecalciferol” OR “25-hydroxyvitamin d” OR “25-hydroxyvitamin d2” OR “25-hydroxyvitamin d3” OR “25-OH-vitamin d” OR “25-OH-vitamin d3” OR hydroxycholecalciferol OR “25(OH)D” OR “1.25(OH)2D” OR “1.25(OH)2D3” OR “1.25 dihydroxyvitamin d” OR “1.25 dihydroxy vitamin d3” OR “1.25 dihydroxyvitamin d3” OR “1.25 dihydroxy vitamin d3” OR “1,25-dihydroxyvitamin d” OR “1,25-dihydroxy vitamin d” OR “1,25-dihydroxyvitamin d3” OR “1,25-Vitamin D3” OR “hypovitaminosis D”)] AND [(“COVID-19” OR “SARS-CoV-2” OR “coronavirus” OR “2019 novel coronavirus infection” OR “2019-nCoV disease” OR “novel coronavirus”)] More information about the search strategy is presented in the Supplementary Material.

### Study Selection

Endnote X9 Program (23) was employed to organize the search results and to identify and exclude duplicate studies. The streamlined list of papers was transferred to Rayyan QCRI reference manager (24) for selection. First, titles and abstracts were screened according to selection criteria listed above. Afterwards, a second selection was carried out, in which the full text of the study was evaluated. Selection was performed independently by peers (CSMP and FL) and cases of disagreement were resolved by consensus or, when necessary, a third reviewer was requested (CB). In the case of studies not retrieved, a librarian collaborated to exhaust the possibilities of obtaining them.

### Data Extraction

A standardized extraction form was developed to map the studies' characteristics. The extracted information included: first author; year of publication; country; aim; type of study; characteristics of the study population (sample size, sex, and age); study duration; authors' affiliation; funding; description of the sponsor's role; declaration of COI; acknowledgments; journal name; journal's impact factor according to Pubmed Impact Factor Chrome Extension.

In order to exhaust the identification of COI situations in the sample, we sought to identify in COI and funding statements offered by the authors in previous works published in 2020 and 2021, as well as unacknowledged relationships with food- and pharmaceutical- industries. COI was identified when authors acknowledged relationships with industries in the food and nutrition area and/or entities linked to them. Funding was understood as sponsorship of the study and/or author(s) by public or private institutions by means of financial or material support. In cases where the information was not available, it was considered that the authors did not report COI. The Google search platform was used to identify the institutions and companies mentioned in the work when necessary. For the analyses of previous work, papers available in the ORCID declared by the authors were investigated.

CPA in selected studies were identified as follows: striking titles (in which the paper's title disagrees, extrapolates or softens the conclusion of the study); adequacy of chosen exposure and outcome regarding the study's aim; positive language regarding vitamin D supplementation in the prevention or treatment of COVID-19 used in the conclusion section; lack of a section or paragraph clearly reporting the study's limitations and biases; absence of divergent points of view or recognition of other possible explanations for the findings; attempts to discredit other studies.

### Data Synthesis and Analysis

A narrative synthesis was initially performed to describe the included studies. Afterward, a descriptive analysis of the COI assessment was conducted. Categorical variables were shown as absolute numbers and frequencies. The relationship between recommendation of vitamin D supplementation in the papers' conclusions and COI presence was assessed using the Odds Ratio (OR) [95% confidence interval (CI)] and the Fisher's Exact Test. A *p*-value < 0.05 was indicative of statistical significance. All analyses were performed using the SPSS software, version 24.0 (IBM Corp., Armonk, NY, USA).

## Results

**Figure 1:**
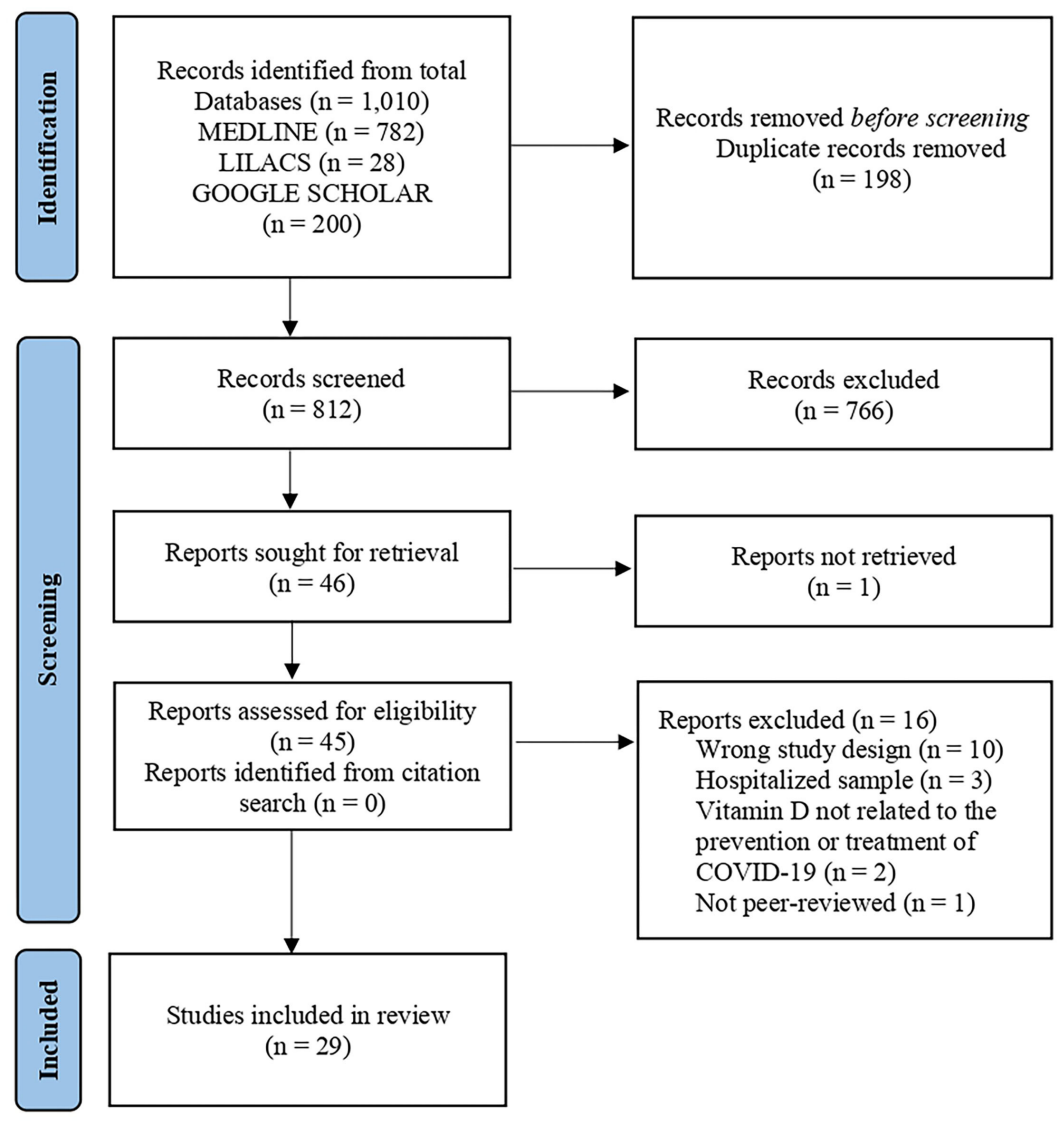
PRISMA study flow diagram for search up to August 24, 2021.

A total of 1,010 articles were found. After duplicates were excluded 812 remained to be screened. After exclusion of results unrelated to the topic, 45 papers were selected for full text analysis, and only one study was not retrieved for a full text analysis. Finally, 29 of which met the inclusion criteria (Figure 1).

### Study Characteristics

Regarding design, of the 29 selected studies, 8 were case-control (25–32), 13 were cohort (33–45), 6 were cross-sectional (46–51) and two were ecological (52, 53). The sample size ranged from 40 to 987,849 participants. The ecological studies by Papadimitriou et al. (53) and Mariani et al. (52) evaluated data from 26 and 46 countries, respectively. The subject's age ranged from 1 month to 95 years of age. Both sexes were evaluated in 28 studies, with the exception of the study by Sinaci et al. (30) which included only females. There was a greater number of publications in *Nutrients* journal (three papers), followed by *JAMA Network Open* (two papers). Impact Factor of these journals are 4.546 and 5.032, respectively. The 29 studies were carried out between the years 2020 and 2021, with twenty published in the year 2021 (25–30, 34–39, 42, 46, 47, 49–53) and nine published in the year 2020 (31–33, 36, 38, 40–42, 48) (Table 1). Of these, 8 studies investigated electronic health record data or biobank data obtained prior to the COVID-19 pandemic (33, 34, 36, 41, 43, 45, 48, 52).

**Table 1:** Study characteristics (*n* = 29).

**References, Country**	**Aim of study**	**Study design**	**Sample characteristics**		**Data collection timeframe**	**Journal**	**Impact factor**
			***n***	**Age (years) Median, mean (SD) or range**			
Abdulateef et al. (46), Iraq	Evaluate COVID-19 severity and to relate them to sociodemographic characteristics and prophylactic dietary supplements	Cross-sectional	428	33	July to August 2020	Open Medicine	1.204
Al-Daghri et al. (25), Saudi Arabia	Determine differences in the serum 25(OH)D concentrations of adult residents screened for SARS-CoV-2 and its association with risk of COVID-19 infection together with other comorbidities	Case-control	220	43 ± 15	May to July 2020	Journal of Translational Medicine	4.124
AlSafar et al. (51), United Arab Emirates	Examine the relation between vitamin D status and COVID-19 severity and mortality	Cross-sectional	464	46.6 ± 14.9	August 2020 to February 2021	Nutrients	4.546
Basaran et al. (26), Turkey	Investigate the relationship between the levels of vitamin D and severity of COVID-19	Case-control	204	57.6 ± 18	NA	Bratislava Medical Journal	1.2
Brenner et al. (33), Germany	Assess the prevalence of vitamin D insufficiency and deficiency and its association with mortality from respiratory diseases during 15 years of follow-up and discuss potential implications for prevention in the context of the ongoing COVID-19 pandemic	Cohort	9,548	50–75	Databases from the years 2000 to 2002	Nutrients	4.546
Elliott et al. (34), United Kingdom	Investigate risk factors for COVID-19 mortality in comparison with non-COVID-19 mortality using data from the community-based UK Biobank	Cohort	502,506	40–69	Database from the years 2006 to 2010	European Journal of Epidemiology	7.135
González-Estevez et al. (47), Mexico	Evaluate the food intake quality of SARS-CoV-2 positive individuals and some of the common factors related to vitamin D deficiency	Cross-sectional	40	43.98 ± 13.65	August to September 2020	International Journal of Environmental Research and Public Health	2.849
Gündüz and Karaaslan (32), Turkey	Compare the vitamin D levels between the group of patients diagnosed with COVID- 19 and healthy controls, and to investigate the relationship between vitamin D levels and clinical findings	Case-control	419	40.4 ± 14.4 (cases), 38.8 ± 15.4 (controls)	NA	Annali Italiani di Chirurgia	0.77
Hastie at al. (41), Scotland	Establish whether blood 25(OH)D concentration was associated with COVID-19 risk	Cohort	348,598	37–73	Databases from 2006 to 2010 and 2020	Diabetes and Metabolic Syndrome: Clinical Research and Reviews	2.38
Israel et al. (27), Israel	Identify whether existing medications have a protective effect against severe disease	Case-control	60,039	18–95	November to December 2020	eLife	7.08
Jude et al. (28), United Kingdom	To examine whether hospitalization with COVID-19 is more prevalent in individuals with lower vitamin D levels	Case-control	80.670	53.2	April 2020 to January 2021	The Journal of Clinical Endocrinology and Metabolism	5.399
Katz et al. (48), USA	Determine the strength of association between vitamin deficiency and COVID-19	Cross-sectional	987,849	NA	Databases from 2015 to 2020	Nutrition	3.639
Kaufman et al. (42), USA	Determine if circulating 25(OH)D levels are associated with SARS-CoV-2 positivity rates with severe acute respiratory disease	Cohort	191,779	54	March to June 2020	PLOS ONE	2.74
Li et al. (43), China	Assess the possible roles of metabolic/obesity phenotypes and vitamin D status in increasing the greater severity of COVID-19	Cohort	353,299	67.7 ± 8	March to May 2020 and databases from 2006 to 2010	Aging & Disease	5.402
Li et al. (44), USA	To examine whether low levels of vitamin D are associated with SARS-CoV-2 seropositivity, an indicator of previous infection	Cohort	18,148	47	December 2020 to March 2021	JAMA Network Open	5.032
Louca et al. (49), United Kingdom	Investigate whether users of the COVID-19 Symptom Study app who regularly took dietary supplements were less likely to test positive for SARS-CoV-2 infection	Cross-sectional	372,720	16–90	July 2020	BMJ Nutrition, Prevention & Health	NA
Luo et al. (50), China	To investigate whether vitamin D deficiency is associated with COVID-19 incidence and disease severity	Cross-sectional	895	56	February to March 2020	The Journal of Nutrition	4.281
Ma et al. (45), USA	Investigate the prospective association between habitual use of vitamin D supplements and risk of COVID-19 infection, and assess whether such an association differed according to the different levels of circulating and genetically predicted vitamin D	Cohort	8,297	37–73	March to June 2020 and databases from 2006 to 2010	The American Journal of Clinical Nutrition	6.766
Mariani et al. (52), Argentina	Assess the association between vitamin D deficiency and COVID-19 incidence, complications, and mortality	Ecological	46 countries	NA	Databases from 2019 and July 2020	Health Security	1.297
Matin et al. (29), Iran	Analyze the role of vitamin D and albumin in the severity, progression, or possible prevention of COVID-19 infection	Case-control	394	NA	July to September 2020	Archives of Microbiology	1.884
Meltzer et al. (35), USA	Elucidate if there are differences in vitamin D levels greater than 30 ng/mL associated with having test results positive for COVID-19	Cohort	4,638	52.8 ± 19.5	March to December 2020	JAMA Network Open	5.032
Oristrell et al. (36), Barcelona	To analyze the associations between cholecalciferol or calcifediol supplementation, serum 25(OH)D levels and COVID-19 outcomes	Cohort	325,029	>18	April 2019 to February 2020	Journal of Endocrinological Investigation	3.397
Papadimitriou et al. (53), Greece	To elucidate the role of vitamin D status in the COVID-19 pandemic	Ecological	26 European countries	NA	June 2020	World Journal Virology	Unknown
Pizzini et al. (36), Austria	To investigate associations of vitamin D status to disease presentation within the CovILD registry	Cohort	109	58 ± 14	April 2020	Nutrients	4.546
Raisi-Estabragh et al. (38), United Kingdom	Examine whether the greater severity of COVID-19 is explained by cardiometabolic, socio-economic or behavioral factors	Cohort	4,510	40–69	March to May 2020	Journal of Public Health	1.806
Ribeiro et al. (39), Brazil	To associate the 25(OH)D concentrations and lipid profile prior to the SARS-CoV-2 tests in a population from a sunny region	Cohort	1,634	45 ± 16	April to December 2020	Clinica Chimica Acta	2.615
Sinaci et al. (30), Turkey	To evaluate the vitamin D status of pregnant women with COVID-19, and the association between vitamin D level and severity of COVID-19	Case-control	491	29.6 ± 5.72 (case) 27.48 ± 5.14 (controls)	July to December 2020	The Journal of Steroid Biochemistry and Molecular Biology	3.813
Ye et al. (31), China	To examine the relationship between serum 25(OH)D level and COVID-19 infection, its severity, and its clinical characteristics	Case-control	142	43	February to March 2020	Journal of the American College of Nutrition	2.297
Yilmaz et al. (40), Turkey	Investigate the prevalence and clinical importance of vitamin D deficiency in children with COVID-19	Cohort	85	1 months−18	March to May 2020	Pediatric Pulmonology	2.534

*SD, standard deviation; NA, not available; 25(OH)D, 25-hydroxyvitamin D*.

### Conflict of Interest Assessment

**Figure 2:**
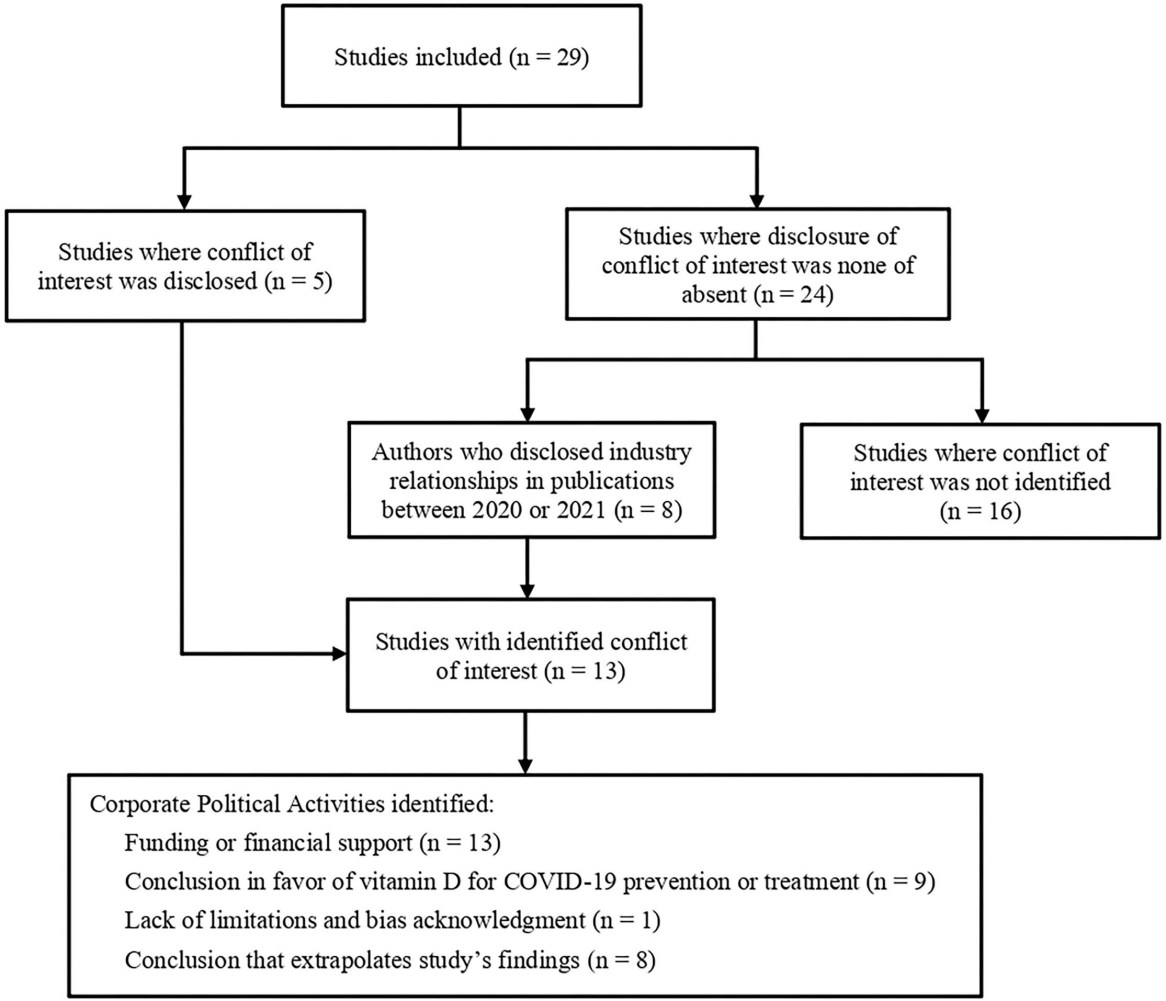
Conflict of Interest and Corporate Political Activities identification flowchart.

Among included studies, five reported COI with industries (42, 44, 49, 51, 53) and 24 reported absence of COI or did not report on COI (25–41, 43, 45–48, 50, 52). After analyzing the funding section as well as other papers published by authors in 2020 and 2021, eight studies were found to have ties with industries (25, 26, 28, 33, 36, 38, 46, 48), totaling 13 studies (44.8% of the sample) where COI was identified. In addition to COI, other CPA were evaluated. No “striking titles” were identified. No attempt to discredit other studies was identified either. Regarding the reporting of limitations and bias, only one study (33) did not objectively report on it. Exposures and outcomes were considered adequate to respond each study's questions. However, in eight of the 29 studies (25, 26, 30–33, 42, 51) authors suggested supplementation of vitamin D for the prevention or treatment of COVID-19 in the conclusion, even though this was not one of the study's stated aims. Of these eight studies which support vitamin D supplementation unsupported by their own data, five (62.5%) were identified as having COI (25, 26, 33, 42, 51). The flowchart of the identification of COI and CPA situations is shown in Figure 2.

Diagnostics-, pharmaceuticals- and food- companies were the source of COI in the analyzed papers. A relationship with diagnostic industry was observed in five of the 13 studies (42, 44, 46, 48, 53), seven of the studies had COI due to their links with the pharmaceutical industry (25, 26, 28, 33, 36, 38, 51), while one study had ties to both pharmaceutical and food industries (49) (Table 2).

**Table 2:** Characterization of conflict of interest present in the sample (*n* = 13).

**References, Country**	**Conclusion in favor of vitamin D supplementation**	**Funding, financial support or employment relationships between authors and corporations**	**Corporation sector**
Abdulateef et al. (46), Iraq	Yes	DA, authored a study supported by the companies MediaMed Lab and Saman Lab in February 2020	Diagnostics
Al-Daghri et al. (25), Saudi Arabia	Yes	AD, Synergy Pharma provided vitamin supplements for the study	Pharmacological
Al Safar et al. (51), United Arab Emirates	Yes	WBG, receives funding from Bio-Tech Pharmacal, Inc. (Fayetteville, AR)	Pharmacological
Basaran et al. (26), Turkey	Yes	TY, had a study funded by Gilead Sciences in January 2021	Pharmacological
Brenner et al. (33), Germany	Yes	HB, had a study funded by Epigenomics, Applied Proteomics and Roche Diagnostics	Pharmacological
Jude et al. (28), United Kingdom	No	EBJ, a study form March 2021 earned him honoraria from the consulting council of Sanofi, had received honoraria as a lecturer from Bayer AG, Boehringer Ingelheim, Eli Lilly, Novo Nordisk and Takeda. In a study published in December 2020, disclosed relationships with Sanofi	Pharmacological
Katz et al. (48), USA	No	JK, disclosed Consulting for HT Bioimaging in a study in October 2020	Diagnostics
Kaufman et al. (42), USA	Yes	HWK, JKN, MHK e CB are directly employed by Quest Diagnostics. HWK, MHK e CB have Quest Diagnostics action shares. MFH is a consultant for Quest Diagnostics and has been a member of the lecturer committee for Abbott Inc. and Hyatt Pharmaceutical Industries Company PLC	Diagnostics
Li et al. (44), USA	No	Study was funded by Quest Diagnostics	Diagnostics
Louca et al. (49), United Kingdom	Yes	TDS, AMV, ERL e SEB consult for Zoe Global Limited ('Zoe'); JW is directly employed by Zoe; PCC has research funding from BASF AS and Bayer Consumer Care, and is a consultant for BASF AS, DSM, Danone Nutricia, Cargill, Smartfish, Nutrileads, Bayer Consumer Care and Pfizer (now GSK) and Consumer Healthcare. He has received refunds for trips and fees conceded by Danone, Fresenius Kabi, Pfizer (now GSK), Consumer Healthcare, Smartfish, Biogredia and the California Walnut Commission. ATC has recied honoraria as a consultant to Bayer Pharma, Pfizer and Boehringer Ingelheim	Pharmacological/ food industry
Oristell et al. (36), Barcelona	Yes	EC, disclosed having received honoraria as a lecturer or consultant from Amgen, Lilly, UCB, Rubió and Theramex in a paper published in 2021. In a different study published 2020 he disclosed to be a lecturer for Amgen Inc., Lilly and Rubió; and receiving honoraria from Stada, Theramex and UCB Pharma	Pharmacological
Papadimitriou et al. (53), Greece	Yes	MFH, was a consultant for Quest Diagnostics and Ontometrics Inc. and a lecturer with Abbott Inc.	Diagnostics
Raisi-Estabragh et al. (38), United Kingdom	No	NH, disclosed in a study published in October 2021 to have received consulting feed, honoraria or subsidies from Alliance for Better Bone Health, Amgen, MSD, Eli Lilly, Servier, Shire, UCB, Consilient Healthcare, Radius Health, Kyowa Kirin, and Internis Pharma	Pharmacological

Only 23% of all COI (3/13) were due to direct funding to carry out the study (39, 41, 49). Authors of two of these studies (39, 41) stated that the sponsoring company did not play a role in the selection or methodological evaluation of the included studies, nor in the interpretation of the results or conclusions reached.

No COI were identified in 16 studies in the sample. Of these, 12 acknowledge funding via grants from governments, universities or academic research centers (25, 27, 31, 33–35, 37, 41, 43, 45, 47, 52) and three studies explicitly stated that there had been no sources of funding and no sponsorships (30, 40, 50). One study did not mention whether or not funding was available (32).

In studies where COI was identified, the chance of a conclusion recommending vitamin D supplementation for the prevention or treatment of COVID-19 was higher than among studies where COI was not identified [OR: 6.75 (1.32, 34.57)]. In the whole sample, most of the studies (16/29, 55.2%) concluded that vitamin D was not associated with the prevention or treatment of COVID-19 (28, 29, 33, 34, 37–41, 43–45, 47, 48, 50, 52). Among independent studies, the prevalence of a lack of association was even higher (12/16, 75.0%). Regarding the studies where COI was identified (25, 26, 33, 36, 42, 46, 49, 51, 53), the majority concluded in favor of an association between vitamin D and prevention or treatment of COVID-19 (9/13, 69.2%, p = 0.027), as described in Table 3.

**Table 3:** Distribution of conclusions regarding vitamin D and COVID-19 prevention or treatment, according to conflict of interest.

**Conflict of interest (*n* = 29)**	**Conclusion regarding vitamin D supplementation**		**Odds ratio (95% CI)**	***p*-value^a^**
	**Positive association [*n* (%)]**	**No association [*n* (%)]**		
Present (*n* = 13)	9 (69.2%)	4 (30.8%)	6.75 (1.32, 34.57)	0.027
Not present (*n* = 16)	4 (25.0%)	12 (75.0%)		

a *Fisher's exact test*.

*CI, confidence interval*.

## Discussion

In the present review, we tracked COI and CPA in observational studies regarding vitamin D and COVID-19. We found that almost half of the studies published between the emergence of the novel coronavirus to August 2021 were potentially conflicted, though most did not state this plainly. There is a dire need for more transparency in the reporting of COI.

The identification of CPA in the present study was based on the proposal by Mialon et al. (12), which focuses on the food industry. Nonetheless, we identified the application of the same strategies by other industries, such as pharmaceutical- and diagnostics- companies. Our findings highlight important points revolving around the participation of industries in scientific production in food and nutrition.

The tobacco industry has become well known for its CPA, and the use of similar activities has been reported by pharmaceutical, food, alcohol, diagnostics and gambling companies (12, 16, 54, 55). The diagnostics industry reported here refers to corporations that produce laboratory tests. Importantly, studies sponsored by such companies in the present sample sought to give visibility to their products and services, such as tests for vitamin D levels and tests for SARS-CoV-2 detection.

Financial incentive, such as the funding of studies and authors that was identified in the present sample, has been proposed as one of the six major categories of CPA (13). Previous studies in other fields have also found an absence of COI disclosures, as well as reports of “no disclosures” by authors who had financial relationships with industries, characterizing omission (56). A recent study that analyzed the evidence supporting global guidelines for vitamin D and calcium recommendations in bone health showed that COI disclosure was low, and studies with absent or unclear COI disclosures were more likely to come to conclusions favorable to vitamin D and/or calcium intake than those with disclosures (57). In our review we found a lack of clear statements regarding the origins of funding, the sponsor's role in the study as well as a lack of bias and limitations in reporting. COI was not always reported straightforwardly in the sample, as we noticed the use of the distinct fields such as “acknowledgments” and “additional information” to inform readers about funding, materials donations, and other types of industry involvement.

Such findings point toward a lack of understanding of what constitutes a COI, or to the presence of insecurity, on the part of authors, about framing their relationships with companies as such. While the way of reporting on COI may also be related to each journal's rules, intentional omission cannot be ruled out. In any case, the absence of this information hinders the identification of COI situations (54) and academic journals and their editorial staff, as co-responsible for this potentially biased scientific production, should demand greater transparency.

Three papers within our sample (42, 44, 53) were financially tied to the company Quest Diagnostics. The studies by Li et al. (44) and Kaufman et al. (42) had similar objectives (examine whether low levels of vitamin D are associated with SARS-CoV-2 positivity) and exposure [total serum 25-hydroxyvitamin D (25(OH)D)]. However, the studies came to different conclusions. Li et al. found that low vitamin D levels were not independently associated with the risk of seropositivity and did not mention supplementation strategies for COVID-19 prevention or treatment. Kaufman et al., on the other hand, concluded that SARS-CoV-2 positivity assessed with nucleic acid amplification testing was strongly associated with circulating levels of 25(OH)D. The authors recommended vitamin D supplementation, even though their study did not tackle this issue. Papadimitriou et al. (53) study has an ecological design, investigating the correlation between published representative-standardized population vitamin D concentrations and several pandemic-related indicators such as total cases per million inhabitants and deaths per million inhabitants in 26 European countries. In finding negative correlations between serious-critical illnesses and deaths and high 25(OH)D concentrations, the authors expressly recommend vitamin D supplementation with the upper tolerable daily doses followed by maintenance doses.

Something in common between two studies mentioned above (42, 53) is the participation of researcher Michael Holick as co-author. Holick has done consulting for and has his work partially funded by several companies which sell supplements, diagnostics, and even tanning beds. He is the author of vitamin D-related books such as 2011's The Vitamin D Solution (58, 59). Recently, one of the studies he co-authored on vitamin D and COVID-19, published in the journal PLoS ONE in 2020, was subject to an “Expression of Concern” by the journal's editorial staff, which brought up a series of methodological problems that called into question the credibility of the study, including the omission of the COI declarations (60). The journal said the study would be re-evaluated, though until the time of writing this manuscript, the study is available for access in the journal (61).

While it is possible to infer several reasons for disagreement between the findings, such as methods used or sample characteristics, for example, it is worth noting that in the two studies that advocate vitamin D supplementation (42, 53) there is one author in common who declares relationships with pharmaceutical industries. Thus, observing the possible interaction of the industry with these studies, CPA can be inferred.

Another noteworthy case is that of William B. Grant. He is co-author of one observational study that made into our selection criteria (51), but most notably is first author of a narrative review that features as a bibliographic reference to most studies analyzed here (62). Published in the journal Nutrients in April 2020, the paper suggested people “at risk of COVID-19” should consider “taking 10,000 IU/d of vitamin D3 for a few weeks to rapidly raise 25(OH)D concentrations, followed by 5,000 IU/d to reduce the risk of infection” (61). This paper had great repercussions, being cited 924 times and featuring as Nutrients' most cited article in the last 2 years and the third most cited of all times, even though its methodology is fragile and the authors conveniently ignored the results of studies that contradicted their thesis (63).

These examples highlight the need to identify COI in scientific production, as well as CPA involving academic publications that gain high repercussions further corporation's market interests, especially in a context of fear and uncertainty such as the COVID-19 pandemic. This review contributes to the qualification of the academic debate surrounding COI. There is an urgent need to denaturalize such relationships, since industries seem to be able to steer research findings according to their interests and drive research agenda in their favor (64), which means, in this case, an incentive to unrestricted supplementation. In clinical practice, this scenario can contribute to an iatrogenic combination of overtesting, overdiagnosis, and overprescription, leading to exposure to overtreatment and overdose (65–68). The management of COI and the creation of policies to mitigate its negative impacts are necessary and have been shown to be beneficial within the area of public health (69).

### Strengths and Limitations

This was a rigorous scoping review on COI in observational studies. As a strength, we went beyond the author's COI declarations, employing CPA framework to identify COI in papers even if it was unacknowledged, which we deem a methodological innovation. Despite Medline being one of the key international general healthcare databases and Lilacs an important regional bibliographic database (70), this study is limited by its design, in that only open access databases were evaluated, since others were inaccessible to the authors when the search was performed. Possible recall bias of selected observational studies and language bias restricted to the language of the descriptors used must be considered.

## Conclusions

It can be concluded that almost half of observational studies linking vitamin D to COVID-19 published before August of 2021 presented COI. Most of them were not disclosed and were only identified after further investigation of CPA in scientific production. The most frequent CPA in our sample was the funding of studies and/or authors. Favorable conclusions were present in most of the studies which had COI, but in the minority of independent studies. Omission of funding statements was common, as well as a lack of disclosure regarding sponsor's role in the study. More studies evaluating COI in biomedical research are needed and more measures must be taken to reduce possible undue influence exerted by these industries in science, clinical practice, and public health.

## Author Contributions

CP, FL, and CB: study concept and design and acquisition and interpretation of data. CP, FL, CB, CC, SR, and MC: drafting of the manuscript. CC, SR, and MC: critical revision of the manuscript for important intellectual content. FL and CB: supervision. All authors have read and agreed to the published version of the manuscript.

## Funding

The Article Processing Charges was funded by the Graduate Program in Food and Nutrition Security (PPGSAN), Federal University of the State of Rio de Janeiro (UNIRIO).

## Conflict of Interest

The authors declare that the research was conducted in the absence of any commercial or financial relationships that could be construed as a potential conflict of interest.

## Publisher's Note

All claims expressed in this article are solely those of the authors and do not necessarily represent those of their affiliated organizations, or those of the publisher, the editors and the reviewers. Any product that may be evaluated in this article, or claim that may be made by its manufacturer, is not guaranteed or endorsed by the publisher.
