# Supplementary material for: Conflict of Interests in the Scientific Production on Vitamin D and COVID-19: A Scoping Review
Source: Front Public Health. 2022 Jul 11;10:821740. doi: 10.3389/fpubh.2022.821740 (PMC9320027; doi:10.3389/fpubh.2022.821740)
Supplement: Supplementary file 1 [file Table_1.DOCX]

Search strategy and date that was performed in the chosen Databases.

| **Database** | Search  (August 24, 2021) |
| --- | --- |
| **Medline** | ("vitamin D"[Title/Abstract] OR "vitamin d2"[Title/Abstract] OR "vitamin d3"[Title/Abstract] OR "cholecalciferol"[Title/Abstract] OR "ergocalciferol"[Title/Abstract] OR "calcitriol"[Title/Abstract] OR "25-hydroxy-cholecalciferol"[Title/Abstract] OR "25-hydroxyvitamin d"[Title/Abstract] OR "25-hydroxyvitamin d2"[Title/Abstract] OR "25-hydroxyvitamin d3"[Title/Abstract] OR "25-OH-vitamin d"[Title/Abstract] OR "25-OH-vitamin d3"[Title/Abstract] OR hydroxycholecalciferol[Title/Abstract] OR "25(OH)D"[Title/Abstract] OR "1,25(OH)2D"[Title/Abstract] OR "1,25(OH)2D3"[Title/Abstract] OR "1,25 dihydroxyvitamin d"[Title/Abstract] OR "1,25 dihydroxy vitamin d3"[Title/Abstract] OR "1,25 dihydroxyvitamin d3"[Title/Abstract] OR "1,25 dihydroxy vitamin d3"[Title/Abstract] OR "1,25-dihydroxyvitamin d"[Title/Abstract] OR "1,25-dihydroxy vitamin d"[Title/Abstract] OR "1,25-dihydroxyvitamin d3"[Title/Abstract] OR "1,25-Vitamin D3"[Title/Abstract] OR “hypovitaminosis D"[Title/Abstract]) AND ("COVID-19"[Title/Abstract] OR "SARS-CoV-2"[Title/Abstract] OR "coronavirus"[Title/Abstract] OR "2019 novel coronavirus infection"[Title/Abstract] OR "2019-nCoV disease"[Title/Abstract] OR "novel coronavirus"[Title/Abstract]) |
| **Lilacs** | ("vitamin D" OR "vitamina D" OR cholecalciferol OR colecalciferol OR "hypovitaminosis D" OR "hipovitaminosis D") AND ("COVID-19" OR "SARS-CoV-2" OR "coronavirus") |
| **Google Scholar** | ("vitamin D" OR cholecalciferol OR “hypovitaminosis D") AND ("COVID-19" OR "SARS-CoV-2" OR "coronavirus") |
